# Supplementary material for: Health insurance in Nigeria: Findings from the People’s Voice Survey
Source: PLOS Glob Public Health. 2026 Aug 26;6(8):e0006948. doi: 10.1371/journal.pgph.0006948 (PMC13514027; doi:10.1371/journal.pgph.0006948)
Supplement: Supplementary file 1 [file pgph.0006948.s001.pdf]

Table S1: Association between insurance, health utilization, and health status (Odds ratios, unweighted logistic regression)

	(1)	(2)	(3)
	Usual source, public primary care	Self-rated health very good/excellent	Any chronic illness
Has insurance	0.479**	1.024	1.209
	(0.141)	(0.143)	(0.235)
Lives in urban area	0.434***	1.024	1.092
	(0.071)	(0.156)	(0.235)
Female	1.518***	1.225*	1.470***
	(0.200)	(0.133)	(0.177)
Higher education	0.790	1.084	0.835
	(0.119)	(0.133)	(0.158)
Age: 30-39	0.993	0.707**	1.391*
	(0.153)	(0.117)	(0.265)
40-49	0.944	0.415***	3.361***
	(0.125)	(0.068)	(0.709)
50-59	0.976	0.385***	4.435***
	(0.219)	(0.078)	(1.182)
60-69	0.583	0.593**	6.134***
	(0.457)	(0.157)	(2.152)
70-79	1.000	0.358	11.054***
	(.)	(0.286)	(8.135)
80+	1.000	0.423	1.000
	(.)	(0.577)	(.)

Middle income	0.620***	1.366***	0.597***
	(0.083)	(0.162)	(0.098)
Highest income	0.467***	1.558***	1.080
	(0.099)	(0.222)	(0.288)
N	2353	2368	2305
Pseudo R^2	0.109	0.044	0.080
Exponentiated coefficients; standard errors in parentheses			
* p<0.10	** p<0.05	*** p<0.01	
