# Supplementary material for: Health insurance in Nigeria: Findings from the People’s Voice Survey
Source: PLOS Glob Public Health. 2026 Aug 26;6(8):e0006948. doi: 10.1371/journal.pgph.0006948 (PMC13514027; doi:10.1371/journal.pgph.0006948)
Supplement: Supplementary file 2 [file pgph.0006948.s002.pdf]

Table S2: Association between insurance, health utilization, and health status (Odds ratios, population-weighted logistic regression)

	(1)	(2)	(3)
	Usual source, public primary care	Self-rated health very good/excellent	Any chronic illness
Has insurance	1.004	0.786	1.326
	(0.704)	(0.286)	(0.611)
Lives in urban area	0.485	0.984	1.637
	(0.222)	(0.307)	(0.799)
Female	2.193**	1.429	1.593
	(0.699)	(0.375)	(0.581)
Higher education	0.657	0.880	0.988
	(0.186)	(0.178)	(0.285)
Age: 30-39	0.574*	0.833	1.038
	(0.184)	(0.264)	(0.338)
40-49	0.905	0.317***	5.673***
	(0.369)	(0.095)	(2.112)
50-59	2.718**	0.340**	8.236***
	(1.105)	(0.154)	(4.850)
60-69	0.344	1.006	7.001***
	(0.468)	(0.514)	(3.769)
70-79	1.000	0.034***	2.627
	(.)	(0.043)	(4.453)
80+	1.000	0.324	1.000
	(.)	(0.674)	(.)

Middle income	0.556	1.579*	0.410**
	(0.217)	(0.417)	(0.169)
Highest income	0.199***	2.407***	0.379**
	(0.078)	(0.677)	(0.166)
N	2353	2368	2305
Pseudo R^2	0.236	0.179	0.220
Exponentiated coefficients; standard errors in parentheses			
* p<0.10	** p<0.05	*** p<0.01	
