# Supplementary material for: Health insurance in Nigeria: Findings from the People’s Voice Survey
Source: PLOS Glob Public Health. 2026 Aug 26;6(8):e0006948. doi: 10.1371/journal.pgph.0006948 (PMC13514027; doi:10.1371/journal.pgph.0006948)
Supplement: Supplementary file 3 [file pgph.0006948.s003.pdf]

Table S3: Association between insurance and preventive screenings (Odds ratios, unweighted logistic regression)

	(1)	(2)	(3)	(4)	(5)
	Blood pressure	Blood sugar	Cholesterol	Eyes checked	Teeth checked
Has insurance	2.147***	2.220***	2.819***	2.190***	2.367***
	(0.415)	(0.474)	(0.423)	(0.285)	(0.491)
Lives in urban area	0.678**	1.082	0.964	1.096	1.068
	(0.110)	(0.149)	(0.150)	(0.206)	(0.192)
Female	1.465**	1.205	1.275	1.222	0.995
	(0.223)	(0.158)	(0.217)	(0.171)	(0.134)
Higher education	1.610***	1.491***	1.410***	1.383*	1.159
	(0.192)	(0.148)	(0.174)	(0.233)	(0.344)
Age: 30-39	1.000	1.000	1.000	1.310***	1.036
	(.)	(.)	(.)	(0.119)	(0.156)
40-49	1.730***	1.536***	1.156	1.683***	0.860
	(0.192)	(0.198)	(0.310)	(0.196)	(0.112)
50-59	3.684***	3.755***	2.689***	3.463***	1.206
	(0.639)	(1.001)	(0.528)	(0.585)	(0.285)
60-69	4.453***	11.761***	6.375***	3.573***	1.689**
	(1.491)	(4.276)	(1.732)	(1.080)	(0.442)
70-79	0.446	1.719	7.220**	3.077*	1.812
	(0.441)	(1.610)	(6.647)	(1.891)	(1.158)
80+	1.000	2.817	3.598	13.048**	1.000

	(.)	(2.909)	(4.016)	(16.732)	(.)
Middle income	1.298*	1.257*	1.073	1.055	0.973
	(0.191)	(0.164)	(0.182)	(0.115)	(0.238)
Highest income	1.512**	1.658***	1.576**	1.297*	1.016
	(0.285)	(0.313)	(0.359)	(0.187)	(0.157)
N	1545	1545	1512	2340	2358
Pseudo R2	0.100	0.118	0.129	0.077	0.048
Exponentiated coefficients; Standard errors in parentheses					
* p<0.10	** p<0.05	*** p<0.01			
