# Supplementary material for: Health insurance in Nigeria: Findings from the People’s Voice Survey
Source: PLOS Glob Public Health. 2026 Aug 26;6(8):e0006948. doi: 10.1371/journal.pgph.0006948 (PMC13514027; doi:10.1371/journal.pgph.0006948)
Supplement: Supplementary file 4 [file pgph.0006948.s004.pdf]

Table S4: Association between insurance and preventive screenings (Odds ratios, population-weighted logistic regression)

	(1)	(2)	(3)	(4)	(5)
	Blood pressure	Blood sugar	Cholesterol	Eyes checked	Teeth checked
Has insurance	1.011	3.957**	1.483	1.792*	2.067*
	(0.592)	(2.182)	(0.477)	(0.636)	(0.862)
Lives in urban area	0.993	0.968	4.287***	1.093	0.871
	(0.328)	(0.372)	(1.811)	(0.550)	(0.309)
Female	1.553	1.322	2.539**	0.941	0.611
	(0.491)	(0.432)	(1.059)	(0.231)	(0.200)
Higher education	1.624**	1.110	1.419	1.680**	0.883
	(0.382)	(0.264)	(0.342)	(0.369)	(0.364)
Age: 30-39	1.000	1.000	1.000	2.061	1.551
	(.)	(.)	(.)	(0.972)	(0.457)
40-49	1.909	2.024***	0.836	1.200	1.608
	(0.960)	(0.544)	(0.537)	(0.391)	(0.744)
50-59	5.358***	5.501***	1.777	4.843***	3.821**
	(2.589)	(2.296)	(1.114)	(2.253)	(2.434)
60-69	11.339***	17.354***	4.503**	2.983*	3.157
	(7.668)	(12.743)	(2.683)	(1.862)	(2.294)
70-79	0.070*	0.199	0.226	0.432	0.104*
	(0.097)	(0.253)	(0.400)	(0.559)	(0.126)
80+	1.000	3.175	24.68***	10.844*	1.000
	(.)	(3.973)	(27.783)	(14.065)	(.)

Middle income	1.743**	0.829	0.990	0.828	0.940
	(0.462)	(0.363)	(0.523)	(0.209)	(0.341)
Highest income	2.375*	1.375	1.594	0.755	1.859
	(1.148)	(0.638)	(0.883)	(0.342)	(0.836)
N	1545	1545	1512	2340	2358
Pseudo R^2	0.270	0.252	0.291	0.139	0.235
Exponentiated coefficients; standard errors in parentheses					
* p<0.10	** p<0.05	*** p<0.01			
