# Supplementary material for: Health insurance in Nigeria: Findings from the People’s Voice Survey
Source: PLOS Glob Public Health. 2026 Aug 26;6(8):e0006948. doi: 10.1371/journal.pgph.0006948 (PMC13514027; doi:10.1371/journal.pgph.0006948)
Supplement: Supplementary file 5 [file pgph.0006948.s005.pdf]

Table S5: Association between Insurance and Facility Visits (Odds ratios, unweighted logistic regression)

	(1)	(2)
	Any in-person visit	In-patient stay
Has insurance	2.442***	2.117***
	(0.321)	(0.301)
Lives in urban area	0.843	0.752*
	(0.106)	(0.118)
Female	1.709***	2.134***
	(0.110)	(0.431)
Higher education	1.376***	1.177
	(0.122)	(0.184)
age 18-29	1.000	1.000
	(.)	(.)
Age: 30-39	1.230*	1.256
	(0.142)	(0.294)
40-49	1.621***	1.300*
	(0.219)	(0.197)
50-59	1.666***	0.822
	(0.285)	(0.219)
60-69	3.332***	1.241
	(1.125)	(0.362)
70-79	1.000	6.699
	(.)	(7.829)
80+	0.223	1.000
	(0.256)	(.)

Middle income	1.255*	1.022
	(0.162)	(0.157)
Highest income	1.554*	1.031
	(0.354)	(0.155)
N	2350	2331
Pseudo R^2	0.070	0.062
Exponentiated coefficients; standard errors in parentheses		
*p<0.10	** p<0.05	*** p<0.01
