# Supplementary material for: Health insurance in Nigeria: Findings from the People’s Voice Survey
Source: PLOS Glob Public Health. 2026 Aug 26;6(8):e0006948. doi: 10.1371/journal.pgph.0006948 (PMC13514027; doi:10.1371/journal.pgph.0006948)
Supplement: Supplementary file 6 [file pgph.0006948.s006.pdf]

Table S6: Association between Insurance and Facility Visits (Odds ratios, population-weighted logistic regression)

	(1)	(2)
	Any in-person visit	Any inpatient stay
Has insurance	2.923***	2.631**
	(1.106)	(1.156)
Lives in urban area	1.413	0.771
	(0.392)	(0.311)
Female	1.808***	3.059***
	(0.405)	(1.001)
Higher education	1.318	1.383
	(0.233)	(0.337)
Age: 30-39	1.466	0.821
	(0.360)	(0.306)
40-49	2.728**	2.387*
	(1.209)	(1.060)
50-59	1.111	0.758
	(0.604)	(0.543)
60-69	2.917	0.787
	(2.274)	(0.638)
70-79	1.000	26.372***
	(.)	(27.638)
80+	0.144*	1.000
	(0.165)	(.)
Middle income	1.230	0.685
	(0.415)	(0.316)

Highest income	1.639*	0.490
	(0.446)	(0.213)
N	2350	2331
Pseudo R^2	0.182	0.177
Exponentiated coefficients; standard errors in parentheses		
* p<0.10	** p<0.05	*** p<0.01
