# Supplementary material for: Health insurance in Nigeria: Findings from the People’s Voice Survey
Source: PLOS Glob Public Health. 2026 Aug 26;6(8):e0006948. doi: 10.1371/journal.pgph.0006948 (PMC13514027; doi:10.1371/journal.pgph.0006948)

Figure S1: Factors associated with having either NHIS or private health insurance (Odds ratios, population-weighted regression)

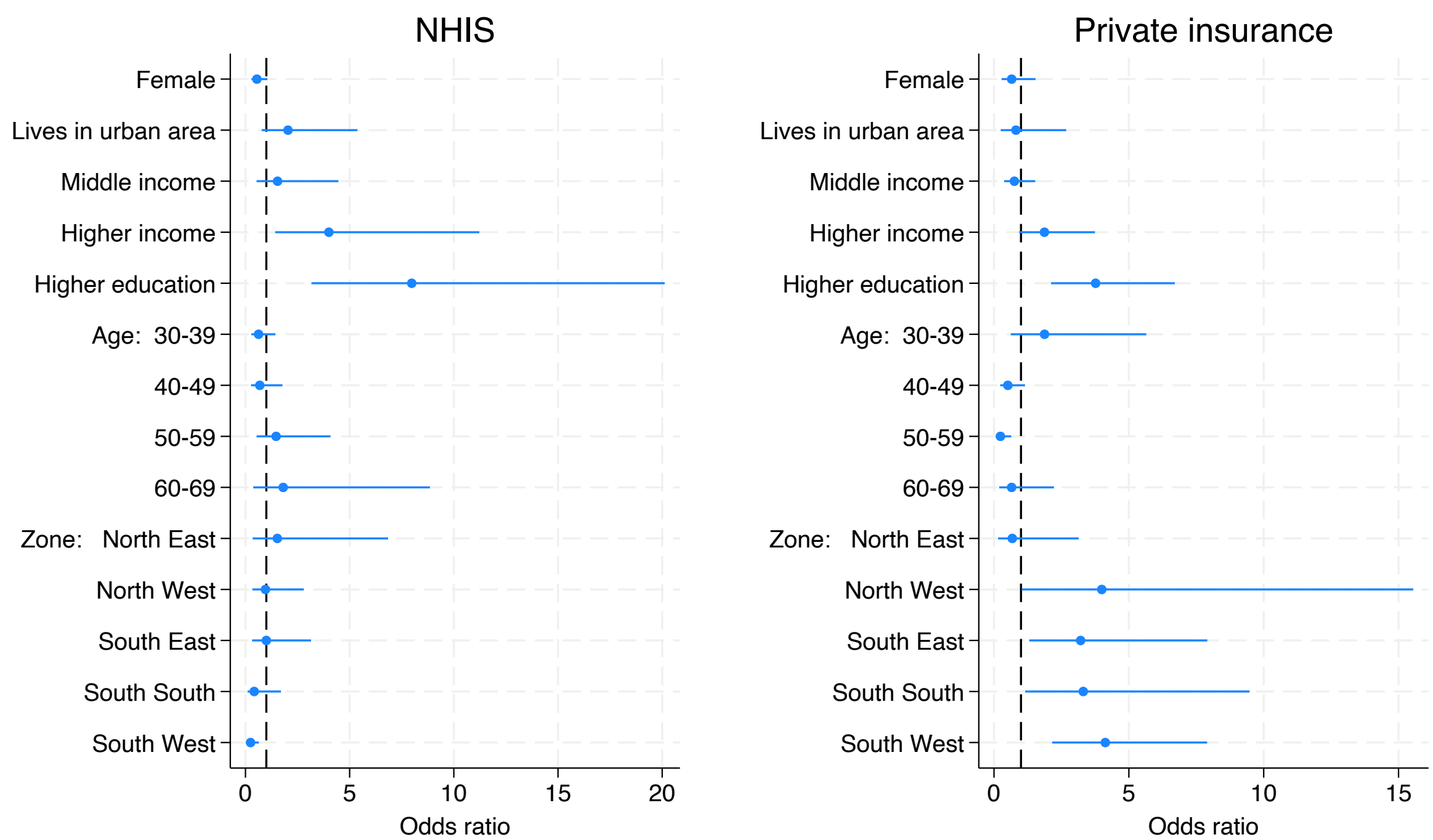

Supplement: Supplementary file 7 [file pgph.0006948.s007.pdf]
